# Evaluation of *Colocasia esculenta* Schott in anti-cancerous properties with proximity extension assays

**DOI:** 10.29219/fnr.v65.7549

**Published:** 2021-10-04

**Authors:** Liang Wu, Yuxuan Wang, Xiaoyan Wang, Jun Liao, Hao Dong, Xiyunyi Cai, Yurong Wang, Harvest F. Gu

**Affiliations:** 1 Jiangsu Key Laboratory of Drug Screening, China Pharmaceutical University, Nanjing, China; 2 Department of Pharmacology, China Pharmaceutical University, Nanjing, China; 3 School of Basic Medicine and Clinical Pharmacy, China Pharmaceutical University, Nanjing, China

**Keywords:** *Colocasia esculenta Schott*, Local cultivar, Oncology, Phytochemistry, Proximity extension

## Abstract

**Background:**

*Colocasia esculenta* Schott (called as Xiangshayu in Chinese) is an excellent local cultivar of the genus polymorpha in Jiangsu Province, China.

**Objective:**

In the present study, we have performed a comparative study before and after dietary consumption with *Colocasia esculenta* Schott to evaluate its anti-cancerous properties.

**Design:**

Forty-two healthy volunteers were recruited, and dietary consumption with 200 g of tap water cooked *Colocasia esculenta* Schott daily was conducted for 1 month. Plasma samples from the subjects before and after dietary consumption with *Colocasia esculenta* Schott were analyzed with proximity extension assays for the alteration of 92 proteins in relation with cancers, while blood samples were examined for physiological parameters with an automatic biochemical analyzer. Bioinformatic analyses were conducted using MalaCards and GEPIA.

**Results:**

After taking dietary consumption with *Colocasia esculenta* Schott, circulating CYR61, ANXA1, and VIM protein levels in the subjects was found to be most significantly downregulated, while for ITGB5, EPHA2, and CEACAM1, it was upregulated. Alternation of these proteins was predicted to be associated with the development of tumors such as pancreatic adenocarcinoma and breast and prostate cancers.

**Conclusion:**

The present study provides evidence that *Colocasia esculenta* Schott, as a healthy food, has anti-cancerous properties. Further investigation of phytochemistry in *Colocasia esculenta* Schott has been taken into our consideration.

## Popular scientific summary

- Colocasia esculenta Schott is a local cultivar of the genus polymorpha in Jingjiang City, Jiangsu Province, China.
- Blood samples collected from volunteers before and after the dietary intervention with Colocasia esculenta Schott are analyzed with proximity extension assays.
- Data demonstrate that Colocasia esculenta Schott, as a healthy food, has anti-cancerous properties.

*Colocasia esculenta* Schott is an annual herbaceous perennial plant. This plant not only is rich in starch, vitamins, minerals, and other nutrients but also has biological properties in metabolism, including antimicrobial, anti-hepatotoxic, anti-cancerous, anti-lipid-peroxidative, anti-melanogenic activities, etc. (1, 2). Furthermore, Park et al. have demonstrated that the polysaccharides extracted from *Colocasia esculenta* Schott have antitumor effects (3). Brown et al. have found that soluble starch extracts made from steamed *Colocasia esculenta* may improve antiproliferative activity on rat YYT colon cancer cell lines and activated lymphocytes from spleen cells (4). Kundu et al. have reported that the water-soluble extract of *Colocasia esculenta* can effectively inhibit the spontaneous metastasis and lung colonization of breast transplanted tumors in ER, PR, and Her-2/neu-negative breast cancer mouse models (5). In Jingjiang area of Jiangsu Province, China, *Colocasia esculenta* Schott, as an excellent local cultivar of the genus polymorpha, is commonly called as ‘Xiangshayu’ in Chinese, which means that *Colocasia esculenta* Schott has fragrant in smell and crisp in taste (6).

Over the last 50 years, the remarkable advances made in life sciences, medicine, diagnostics, forensics, and biotechnology are inconceivable without the contributions from two key technologies: polymerase chain reaction (PCR) for the detection of nucleic acids and antibody-based methods for the detection of proteins. Proximity extension assays (PEA) are developed based on the combination of these key technologies and become the immunoassay for the detection of protein molecules via DNA ligation and amplification, offering high specificity and sensitivity (7–9). Therefore, PEA is currently the most powerful immunoassay for the detection of the protein molecules via DNA ligation and amplification.

In the current study, we applied the advantages of PEA to analyze the alteration of 92 proteins in relation with cancers for the healthy subjects before and after dietary intervention with *Colocasia esculenta* Schott. The purpose of this study was to analyze the biological properties of *Colocasia esculenta* Schott from Jingjiang city, Jiangsu Province, China, in relation with oncology.

## Materials and methods

### Subjects

A total of 42 volunteers (19 males and 23 females) were recruited for this study. They were either university or postgraduate students and at the age between 20 and 35 years old. All subjects had an annual healthy examination in university hospital, and no disease in stomach, liver, and kidney was recorded. Physiological characteristics of all subjects are summarized in Table 1. The subjects with any criteria, including 1) liver and renal function insufficiency (referring to aspartate aminotransferase >1 times the upper limit of normal and creatinine >1.2 times the upper limit of normal, respectively), 2) alcohol abuse, asthma, hyperthyroidism, malignancy, and other endocrine diseases, 3) a surgery within the recent 2 months; d) hypertension (blood pressure >140/90 mmHg), 4) suffering from biliary, intestinal, pancreatic, and other infection diseases, were excluded in this study.

**Table 1:** Physiological parameters of the subjects

*N*	All subjects	Males	Females

	41	19	22
Age (years)	23.88±1.72	23.95±2.17	23.82±1.26
Body mass index (kg/m^2^)	21.26±2.19	21.54±2.30	21.03±2.12
Waist (cm)	75.49±6.67	79.84±6.14	71.73±4.52
Waist-to-hip ratio	0.75±0.05	0.77±0.06	0.74±0.03
Systolic blood pressure (mmHg)	106.27±11.53	112.37±10.15	101.00±10.12
Diastolic blood pressure (mmHg)	69.88±9.31	74.53±8.66	65.86±8.03
Heart rate (bpm)	78.85±8.46	80.74±10.09	77.23±6.56
Basal metabolic rate (kcal)	1,378.15±165.63	1,529.37±112.99	1,247.55±52.44
Body weight out of fat (kg)	47.63±7.80	54.16±5.59	42.00±4.17
Body fat rate (%)	20.71±5.90	16.36±5.06	24.47±3.53
Total body fat (kg)	12.46±4.03	10.86±4.28	13.84±3.30
Visceral fat (kg)	1.29±0.51	1.34±0.64	1.25±0.36
Subcutaneous fat (kg)	11.17±3.59	9.52±3.64	12.59±2.94
Body moisture content (kg)	34.29±5.62	38.99±4.03	30.24±3.01
Muscle mass (kg)	44.15±7.34	50.36±5.18	38.78±3.80
Protein (kg)	9.87±1.75	11.49±1.07	8.55±0.80
Inorganic salt (kg)	3.50±0.51	3.84±0.43	3.21±0.39

Data were expressed as mean ± SEM.

The current study was approved by the local ethical committee in the affiliated Zhongda Hospital, Southeast University, China, and the IBR number is 2016ZSDYLL075-P01. An informed consent was obtained from all subjects before the study began.

## Study design

**Fig. 1:**
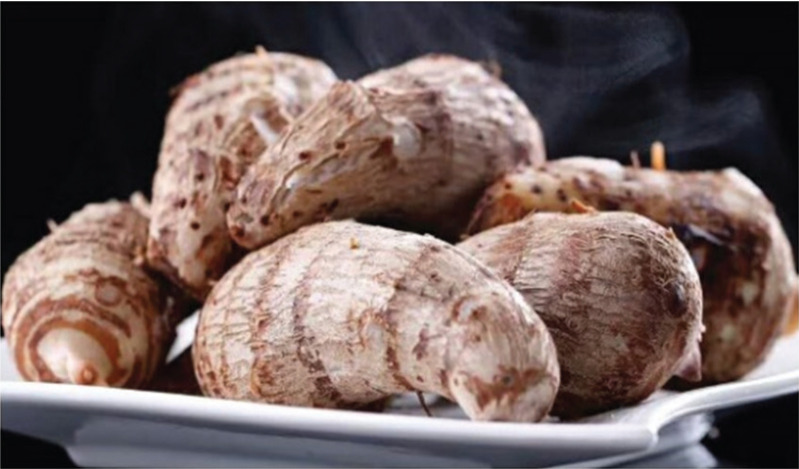
The cooked *Colocasia esculenta* Schott (Xiangshayu). *Colocasia esculenta* Schott was cooked with boiling tap water for 30 min.

The dietary consumption program required all subjects, except for replacing part of their staple foods with *Colocasia esculenta* Schott, to be remained unchanged in their lifestyles and eating habits. Therefore, smoking and alcohol drinking was not allowed in prior to 6 weeks period and during the program. In whole study program, all participates consumed 200 g of tap water cooked *Colocasia esculenta* Schott (for 30 min, as seen in Fig. 1) for breakfast continuously for 30 days. No additional food was included in breakfasts during the study period. Information concerning the nutritional components in *Colocasia esculenta* Schott is represented in Table 2.

**Table 2:** Nutritional components in *Colocasia esculenta* Schott (Xiangshayu)

Item	Amount
Water (%)	≤77
Protein (%)	≥2
Fat (%)	≤0.08
Ash (%)	≤1.21
Fiber (%)	≥0.33
Starch (%)	≥18
Vitamin B1 (mg/kg)	≥0.62
Vitamin C (mg/kg)	≥42
Ca (mg/kg)	≥1.50
Fe (mg/kg)	≥7.80

## Questionnaires

All questionnaires were surveyed and recorded by the trained investigators. The dietary survey tables and notes were explained in detail. The integrity of the questionnaires was reviewed on site to ensure the accuracy and completeness of the original data.

## Determination of physiological parameters

Body height, weight, waist circumference, hip circumference, heart pulse, blood pressures, body composition, and basal metabolic rate of all subjects were measured by professional nurses under the morning fasting state. Body mass index (BMI, kg/m^2^) is commonly used for the evaluation of the nutritional status of adults over 18 years of age, and the formula is BMI = weight (kg)/height (m^2^). Body composition was analyzed using a five-factor method (age, sex, weight, height, and impedance) and a four-pole eight-point contact electrode method.

## Blood sampling

On the first day before the program began and on the last day (the 31st day) after the program ended, physiological and biochemistry parameters of all subjects were measured, respectively. In the meanwhile, anticoagulant and non-anticoagulant blood samples (5 mL each and fasted for 12 h in advance) were collected. The anticoagulant blood samples were used for plasma extraction, while non-anticoagulant blood samples were used for serum extraction. All plasma and serum samples were stored at –80°C and then evaluated before used for analyses. The sample used for analysis was not a pool plasma.

## Proximity extension assays

The experiments for analyses of the plasma samples before and after *Colocasia esculenta* Schott treatment were performed with the PEA protocol (7–9). The assay was Olink Oncology II (Olink Proteomics, Uppsala, Sweden). The probes for PEA were prepared using SMCC ([*N*-Maleimidomethyl] cyclohexane-1-carboxylic acid succinimidyl ester). Two paired antibodies (each with target antigen specificity) were coupled to unique oligonucleotides (A and B), each oligonucleotide having a combination of universal amplification primers and template location of specific detection primers. One site was used for paired annealing between oligonucleotides A and B, and another site was used for binding to molecular beacons and qPCR detection. The performance of all specific qPCR primers has been evaluated by their amplification efficiency. Totally, 92 targeted proteins for analyses were included in the PEA experiments, and all the studied proteins are listed in the Supplemental Table 1. The PEA experiments in the steps of extension and detection were conducted using the instruments, including Verity 96 well Thermal Cycler (ThermoFisher Scientific, MA, USA) and Fluidigm Biomark HD system (Fluidigm, CA, USA).

## Bioinformatical analysis

Biopython was used for bioinformatical analysis. This program includes the modules for reading and writing different sequence file formats and multiple sequence alignments, dealing with 3D macro-molecular structures, interacting with common tools such as BLAST, ClustalW, and EMBOSS, and accessing key online databases (Version 3.6.8, www.biopython.org) (10). MalaCards is an integrated searchable database of human maladies and their annotations, modeled on the architecture and richness of the popular GeneCards database of human genes. MIFTS (MalaCards InFormaTion Scores) are defined as the richness of information in each card and assigned to each disease by summing the base 10 logarithms of the counts of its populated annotations. This score currently ranges from 1 to 101, with the MalaCard scored 101 being the most annotated card. The Gene Expression Profiling Interactive Analysis (GEPIA) is an interactive web application and can demonstrate the differentially displayed expression of the genes between cancer and normal tissues (https://gepia.cancer-pku.cn/) (11). Biovista is a pioneer of systematic drug repositioning, in which we used Vizit for the prediction of connections and networks among the studied proteins (https://www.biovista.com/vizit) based on the references from PubMed (https://www.ncbi.nlm.nih.gov/pubmed) (12, 13). We, thus, used these databases not only for analyzing significantly changed tumor-associated proteins and the related cancers but also for investigating the survival rate of the patients with the studied cancers in terms of the studied gene expression reregulation.

## Statistical analysis

Statistical analysis was performed using the PASW (Version 22.0) and R software (Version 3.6.0). The statistical power on the size of subjects was tested prior to the experiments. Thereafter, the analyses of quantitative data were performed using the paired t-test for normally distributed parameters, while the Wilcoxon test of ranks was alternatively used for traits with non-normal distributions. OPLS-DA (Orthogonal Partial Least Squares Discrimination Analysis) analysis was performed by R statistical analysis software. All data are expressed as means or percentages with standard errors. *P*-value less than 0.05 was considered significant.

## Results

### Altered expression of proteins analyzed with PEA oncology II assays

**Fig. 2:**
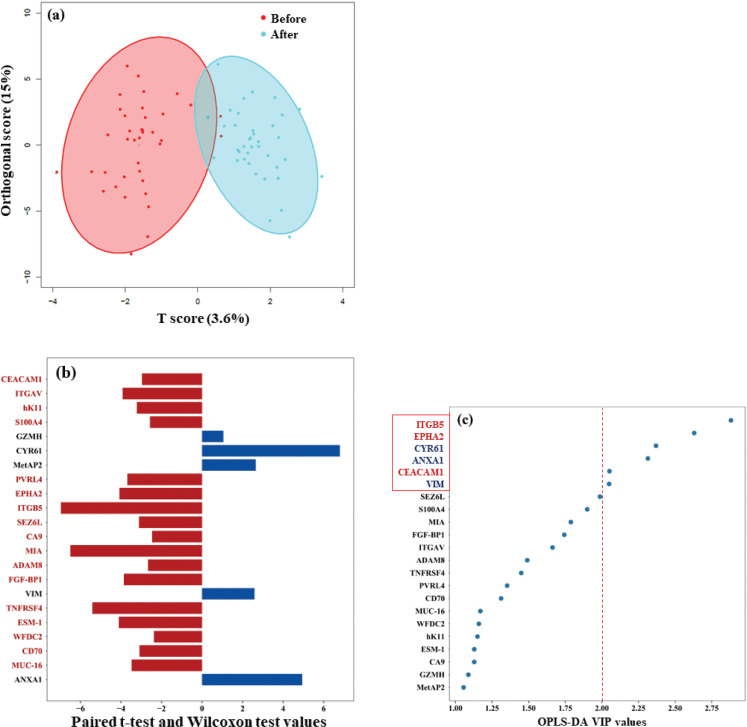
Altered expression of proteins in all subjects before and after *Colocasia esculenta* Schott dietary intervention. a. The 92 studied proteins are clustered before and after *Colocasia esculenta* Schott dietary intervention along the tp1 direction based on the analyses with OPLAS-DA. b. Expression levels of 22 proteins after the *Colocasia esculenta* Schott consumption are alternated significantly. Among them, 17 proteins were upregulated (the protein names were highlighted in red), while five proteins were downregulated (in blue). c. The top six proteins according to OPLAS-DA VIP value at 2.00 are CYR61, ANXA1, and VIM downregulated (*P* = 5.26E–08, 1.22E–04, and 0.0025, respectively) and ITGB5, EPHA2, and CEACAM1 upregulated (*P* = 3.24E–08, 2.30E–04, and 7.68E–06, respectively).

By using PEA oncology II assays, a total of 92 proteins associated with the development of cancer were analyzed in the plasma samples collected from all subjects before and after *Colocasia esculenta* Schott dietary intervention. As observed in the score plots from OPLS-DA analysis, the studied 92 proteins were found to be clustered in the baseline and after *Colocasia esculenta* Schott dietary intervention along the tp1 direction (Fig. 2a). Of the studied 92 proteins, the expression levels of 22 (23.9%) proteins after the *Colocasia esculenta* Schott consumption were found to be significantly changed, that is, 17 proteins were upregulated (the protein names were highlighted in red), while five proteins were downregulated (in blue) (Fig. 2b). Furthermore, top six proteins were selected according to OPLAS-DA VIP value at 2.00. Among these six proteins, cysteine-rich angiogenic inducer 61 (CYR61), annexin A1 (ANXA1), and vimentin (VIM) were found to be downregulated (*P* = 5.26E–08, 1.22E–04, and 0.0025, respectively), while integrin subunit beta 5 (ITGB5), H receptor A2 (EPHA2), and CEA cell adhesion molecule 1 (CEACAM1) were upregulated (*P* = 3.24E–08, 2.30E–04, and 7.68E–06) (Fig. 2c). Data analyses in male and female subjects were done, and the results were attached in Supplemental Fig. 1a and b, respectively.

### Association of altered proteins with cancers predicted with MIFTS and GEPIA

The association of all 22 alternated proteins after dietary intervention with *Colocasia esculenta* Schott was analyzed by using the programs of MIFTS. According to MIFTS, the hits of cancers were listed in Table 3, which implicated that *Colocasia esculenta* Schott might have antitumor effects. Further analyses with GEPIA and Biovista/vizit programs were performed and data were summarized in Supplemental Fig. 3. Several cancers, including lymphoid neoplasm diffuse large B-cell lymphoma, glioblastoma multiforme (GBM), pancreatic adenocarcinoma (PAAD), thymoma, cervical squamous cell carcinoma and endocervical adenocarcinoma, kidney renal clear cell carcinoma, testicular germ cell tumors, thyroid carcinoma, brain lower grade glioma, liver hepatocellular carcinoma, skin cutaneous melanoma, and acute myeloid leukemia (AML), were found to be associated with increased ANXA1, CYR61, and VIM expressions, while all these three protein alternations were associated with PAAD (Supplemental Fig. 2a, b, and c). The decreased expression of CEACAM1 (Supplemental Fig. 2d) and EPHA2 (Supplemental Fig. 2e) was found to be associated with several cancers, but ITGB5 reduction in expression was associated with acute myeloid leukemia (LAML) only (Supplemental Fig. 2f).

**Table 3:** Effects of dietary intervention with *Colocasia esculenta* Schott in relation with oncology

Symbol	*f*	*P*	MIFTS (VIP)	Hits of diseases in MalaCards
CYR61	6.7992	5.26E–08	2.3695	Breast cancer (100/0.225); prostate cancer (93/0.178)
ANXA1	4.9386	1.71E–05	2.3137	Breast Cancer (100/5.447); rheumatoid arthritis (81/4.813); esophageal cancer (82/4.169); lung cancer susceptibility 3 (79/3.404)
VIM	2.5868	0.0097	2.0493	CATARACT 30, MULTIPLE TYPES (36/3.253); malignant peripheral nerve sheath tumor (60/2.483)
MetAP2	2.6473	0.0118	1.0554	Microsporidiosis (44/5.261)
HGF	2.3287	0.0254	1.0090	Breast cancer (100/0.615); hepatocellular carcinoma (96/0.625); lung cancer (99/0.525)
ITGB5	–6.9563	3.24E–08	2.8816	Arrhythmogenic right ventricular cardiomyopathy (59/3.880); osteopetrosis, autosomal dominant 2 (55/3.880); villous adenocarcinoma (34/3.880)
MIA	–6.4936	1.35E–07	1.7877	Melanoma (63/8.505); skin melanoma (67/4.915); gastrointestinal defects and immunodeficiency syndrome (38/4.306)
TNFRSF4	–5.4067	3.99E–05	1.4489	Immunodeficiency 16 (21/7.843); systemic lupus erythematosus (88/4.502)
EPHA2	–4.0746	0.0002	2.6304	Cataract 6, multiple types (33/6.513); adenocarcinoma (70/4.694); ovarian cancer (83/4.292); gastric adenocarcinoma (66/4.136)
CEACAM1	–2.9608	0.0003	2.0517	Leukemia (72/0.825); lung cancer (99/0.088); breast cancer (100/0.749)
ITGAV	–3.9147	0.0004	1.6628	Breast cancer (100/0.466); leukemia (72/0.422); melanoma (62/0.389)
FGFBP1	–3.8539	0.0005	1.7433	Breast cancer (100/0.864); leukemia (72/0.699); lung cancer (99/0.603)
PVRL4	–3.6800	0.0007	1.3528	Breast cancer (100/0.606); adenocarcinoma (70/0.445); ovarian cancer (83/0.440); gastric cancer (78/0.332)
SEZ6L	–3.1062	0.0019	1.9868	Breast cancer (100/0.393); seizure disorder (61/0.396); epilepsy (72/0.369)
MUC16	–3.4749	0.0013	1.1703	Breast cancer (100/0.591); leukemia (72/0.733); adenocarcinoma (70/706)
CD70	–3.0818	0.0039	1.3116	Lymphoproliferative syndrome 3 (29/11.281); systemic lupus erythematosus (88/3.858); leukemia, chronic lymphocytic (78/3.756)
S100A4	–2.5660	0.0103	1.8997	Breast cancer (100/5.808); colorectal cancer (99/6.640); pancreatic cancer (82/5.251); thyroid cancer (72/5.208); gastric adenocarcinoma (66/3.931)
ADAM8	–2.6609	0.0115	1.4900	Asthma (81/5.705); retinitis pigmentosa 7 (40/2.786)
WFDC2	–2.3710	0.0231	1.1599	Ovarian Cancer (83/6.244); prostate cancer (93/0.117)
hK11	–3.2146	0.0027	1.1502	Ovarian Cancer (83/0.127)
ESM1	–4.1003	0.0002	1.1282	Hypertension, Essential (81/4.324); squamous cell carcinoma (58/0.173); oral squamous cell carcinoma (48/0.122)
CA9	–2.4576	0.0188	1.1280	Breast cancer (100/4.989); lung cancer (99/5.388); clear cell renal cell carcinoma (49/4.192); hypoxia (63/3.755)
GZMH	–2.3618	0.0236	1.0883	Cutaneous leishmaniasis (68/0.138)
TNFRSF19	–2.3419	0.0247	1.0079	Ovarian cancer (83/3.014); colorectal cancer (99/0.100)

Comparison analyses from baseline to after dietary intervention with Xiangsha Taro were done by using a. pairwise t-test, b. Log10 pairwise t-test, and c. Wilcoxon test. List of symbols of proteins in oncology is prepared according to *P*-values and VIP scores. Information concerning the hits of diseases is collected from Human Disease Databases named as MalaCards (MIFTS/Score). ADAM8: ADAM metallopeptidase domain 8; ANXA1: annexin A1; CA9: carbonic anhydrase 9; CYR61: cellular communication network factor 1 also known as CCN1 or IGFBP10; CD70: CD70 molecule; CEACAM1: carcinoembryonic antigen-related cell adhesion molecule 1; EPHA2: EPH receptor A2; ESM1: endothelial cell specific molecule 1; HGF: hepatocyte growth factor; hK11: kallikrein-related peptidase 11; ITGAV: integrin subunit alpha V; FGFBP1: fibroblast growth factor binding protein 1; GZMH: granzyme H; ITGB5: integrin subunit beta-5; MIA: MIA SH3 domain containing; MetAP2: methionyl aminopeptidase 2; MUC16: mucin 16, cell surface associated; PVRL4: nectin cell adhesion molecule 4; S100A4: S100 calcium binding protein A4; SEZ6L: seizure related 6 homolog like; TNFRSF4: TNF receptor superfamily member 4; TNFRSF19: TNF receptor superfamily member 19; VIM: vimentin; WFDC2: WAP four-disulfide core domain 2.

## Discussion

In the present study, we have analyzed the antidiabetic properties of *Colocasia esculenta* Schott. We have used PEA, which offers high specificity and sensitivity for protein analysis (7–9, 14), and found that 22 of 92 proteins were alternated after the dietary consumption with *Colocasia esculenta* Schott. Of these 22 alternated proteins, CEACAM1, EPHA2, and ITGB5 were found to be most significantly upregulated, while CYR61, ANXA1, and VIM were most significantly downregulated.

CYR61 (cellular communication network factor 1, also known as CCN1) is involved in the transduction of growth factor and hormone signaling. Several studies have demonstrated that the CYR61 expression is correlated with worse prognosis of breast, colorectal, and prostate cancers (15–17). ANAX1 is a calcium-binding protein involved in arachidonic acid metabolism and epidermal growth factor receptor tyrosine kinase pathway. This protein expression has been implicated in early squamous cell carcinogenesis of esophagus and frequent in esophageal and esophagogastric junction adenocarcinomas (18). VIM is a type III intermediate filament protein and promotes cell migration (19). The expression of VIM in colonic neoplastic cells is found to be correlated with the stage of neoplastic progression, which suggests that VIM is a key factor integrating epithelial to mesenchymal transition, and colonic neoplastic progression in colorectal cancer (CRC) (20). As described briefly from these studies, GYR61, ANAX1, and Vim are mainly concerned in breast, colorectal, and prostate cancers. CEACAM1 is an extensively studied cell surface molecule with established functions in modulating the immune responses associated with infection, inflammation, and cancer (21). CEACAM1 is an extensively studied cell surface molecule with established functions in multiple cancer types, as well as in various compartments of the immune system and plays a dual role in different cancers. Due to its multifaceted role as a recently appreciated immune checkpoint inhibitor and tumor marker, CEACAM1 is an attractive target for cancer immunotherapy (22, 23). EPHA2 (erythropoietin-producing hepatocellular receptor A2) controls multiple physiological processes to maintain homeostasis in normal cells. In many types of solid tumors, it plays a critical role in oncogenic signaling (24, 25). ITGB5 encodes a beta subunit of integrin, which can combine with different alpha chains to form a variety of integrin heterodimers. This molecule can serve as a predictive biomarker for GBM patient survival and is a potential therapeutic target in GBM treatment (26). As one of the mesenchymal markers, ITGB5 may serve as an indicator of the metastatic potential and tumor chemosensitivity (27). Furthermore, data from bioinformatical analyses have implicated that ANAX1, CYR61, and VIM are mainly associated with the development of PAAD and GBM, while CEACAM1 and EPHA2 are associated with adrenocortical carcinoma. However, ITGB5 is found to be associated with LAML.

There is a limitation in the present study because no major chemical component in *Colocasia esculenta* Schott is included in analyses, and no patient with cancers is recruited in clinical trial. Recently, Pereira et al. have demonstrated that tarin is extracted from *Colocasia esculenta* Schott and exhibits recognized biocide activities against viruses and insects. Therefore, tarin, as a GNA-related lectin, may have the potential prophylactic and therapeutic actions on hematopoietic and cancer cells, as a potential immunomodulator (28).

In conclusion, the current study provides evidence that *Colocasia esculenta* Schott is a heathy food and has anti-cancerous biological properties. Further investigation in basic research of chemical compounds such as tarin in *Colocasia esculenta* Schott and clinical trial for patients with cancers has been taken into our consideration.

## Supplementary Material

Evaluation of Colocasia esculenta Schott in anti-cancerous properties with proximity extension assays

## Data Availability

Data are contained within the article.
