# Supplementary material for: Evaluation of Colocasia esculenta Schott in anti-cancerous properties with proximity extension assays
Source: Food Nutr Res. 2021 Oct 4;65:10.29219/fnr.v65.7549. doi: 10.29219/fnr.v65.7549 (PMC8634378; doi:10.29219/fnr.v65.7549)

Supplemental Table 1 A list of proteins included for experimental analyses with proximity extension assays.

No	Protein Name	ID
1	5'-nucleotidase (5'-NT)	P21589
2	A disintegrin and metalloproteinase with thrombospondin motifs 15 (ADAM-TS 15)	Q8TE58
3	Alpha-taxilin (TXLNA)	P40222
4	Amphiregulin (AR)	P15514
5	Annexin A1 (ANXA1)	P04083
6	Carbonic anhydrase 9 (CA9)	Q16790
7	Carboxypeptidase E (CPE)	P16870
8	Carcinoembryonic antigen-related cell adhesion molecule 1 (CEACAM1)	P13688
9	Carcinoembryonic antigen-related cell adhesion molecule 5 (CEACAM5)	P06731
10	Cathepsin L2 (CTSV)	O60911
11	CD27 antigen (CD27)	P26842
12	CD48 antigen (CD48)	P09326
13	CD70 antigen (CD70)	P32970
14	CD160 antigen (CD160)	O95971
15	Cornulin (CRNN)	Q9UBG3
16	C-type lectin domain family 4 member K (CD207)	Q9UJ71
17	C-X-C motif chemokine 13 (CXCL13)	O43927
18	Cyclin-dependent kinase inhibitor 1 (DKN1A)	P38936
19	Delta-like protein 1 (DLL1)	O00548
20	Disintegrin and metalloproteinase domain-containing protein 8 (ADAM 8)	P78325
21	Endothelial cell-specific molecule 1 (ESM-1)	Q9NQ30
22	Ephrin type-A receptor 2 (EPHA2)	P29317
23	FAS-associated death domain protein (FADD)	Q13158
24	Fc receptor-like B (FCRLB)	Q6BAA4
25	Fibroblast growth factor-binding protein 1 (FGF-BP1)	Q14512
26	Folate receptor alpha (FR-alpha)	P15328
27	Folate receptor gamma (FR-gamma)	P41439
28	Furin (FURIN)	P09958
29	Galectin-1 (Gal-1)	P09382
30	Glypican-1 (GPC1)	P35052
31	Granzyme B (GZMB)	P10144
32	Granzyme H (GZMH)	P20718
33	Hepatocyte growth factor (HGF)	P14210
34	ICOS ligand (ICOSLG)	O75144
35	Insulin-like growth factor 1 receptor (IGF1R)	P08069
36	Integrin alpha-V (ITGAV)	P06756

37	Integrin beta-5 (ITGB5)	P18084
38	Interferon gamma receptor 1 (IFN-gamma-R1)	P15260
39	Interleukin-6 (IL-6)	P05231
40	Kallikrein-8 (hK8)	O60259
41	Kallikrein-11 (hK11)	Q9UBX7
42	Kallikrein-13 (KLK13)	Q9UKR3
43	Kallikrein-14 (hK14)	Q9P0G3
44	Ly6/PLAUR domain-containing protein 3 (LYPD3)	O95274
45	Melanoma-derived growth regulatory protein (MIA)	Q16674
46	Mesothelin (MSLN)	Q13421
47	Methionine aminopeptidase 2 (MetAP2)	P50579
48	MHC class I polypeptide-related sequence A/B (MIC-A/B)	Q29983, Q29980
49	Midkine (MK)	P21741
50	Mothers against decapentaplegic homolog 5 (MAD homolog5)	Q99717
51	Mucin-16 (MUC-16)	Q8WXI7
52	Nectin-4 (PVRL4)	Q96NY8
53	Pancreatic prohormone (PPY)	P01298
54	Podocalyxin (PODXL)	O00592
55	Pro-epidermal growth factor (EGF)	P01133
56	Protein CYR61 (CYR61)	O00622
57	Protein S100-A11 (S100A11)	P31949
58	Protein S100-A4 (S100A4)	P26447
59	Proto-oncogene tyrosine-protein kinase receptor Ret (RET)	P07949
60	Receptor tyrosine-protein kinase erbB-2 (ERBB2)	P04626
61	Receptor tyrosine-protein kinase erbB-3 (ERBB3)	P21860
62	Receptor tyrosine-protein kinase erbB-4 (ERBB4)	Q15303
63	R-spondin-3 (RSPO3)	Q9BXY4
64	Secretory carrier-associated membrane protein 3 (SCAMP3)	O14828
65	Seizure 6-like protein (SEZ6L)	Q9BYH1
66	SPARC (SPARC)	P09486
67	Stem cell factor (SCF)	P21583
68	Syndecan-1 (SYND1)	P18827
69	T-cell leukemia / lymphoma protein 1A (TCL1A)	P56279
70	TGF-beta receptor type-2 (TGFR-2)	P37173
71	Tissue factor pathway inhibitor 2 (TFPI-2)	P48307
72	T-lymphocyte surface antigen Ly-9 (LY9)	Q9HBG7
73	Toll-like receptor 3 (TLR3)	O15455
74	Transforming growth factor alpha (TGF-alpha)	P01135
75	Transmembrane glycoprotein NMB (GPNMB)	Q14956
76	Tumor necrosis factor ligand superfamily member 6 (FASLG)	P48023
77	Tumor necrosis factor ligand superfamily member 10 (TNFSF10)	P50591

78	Tumor necrosis factor ligand superfamily member 13 (TNFSF13)	O75888
79	Tumor necrosis factor receptor superfamily member 4 (TNFRSF4)	P43489
80	Tumor necrosis factor receptor superfamily member 6B (TNFRSF6B)	O95407
81	Tumor necrosis factor receptor superfamily member 19 (TNFRSF19)	Q9NS68
82	Tyrosine-protein kinase ABL1 (ABL1)	P00519
83	Tyrosine-protein kinase Lyn (LYN)	P07948
84	WAP four-disulfide core domain protein 2 (WFDC2)	Q14508
85	Vascular endothelial growth factor A (VEGF-A)	P15692
86	Vascular endothelial growth factor receptor 2 (VEGFR-2)	P35968
87	Vascular endothelial growth factor receptor 3 (VEGFR-3)	P35916
88	VEGF-co regulated chemokine 1 (CXL17)	Q6UXB2
89	Vimentin (VIM)	P08670
90	Wnt inhibitory factor 1 (WIF-1)	Q9Y5W5
91	WNT1-inducible-signaling pathway protein 1 (WISP-1)	O95388
92	Xaa-Pro aminopeptidase 2 (XPNPEP2)	O43895

Supplemental Figure 1 Up- and down-regulations of the studied proteins after diet intervention with *Colocasia esculenta* Schott in male (A) and female subjects (B).

A

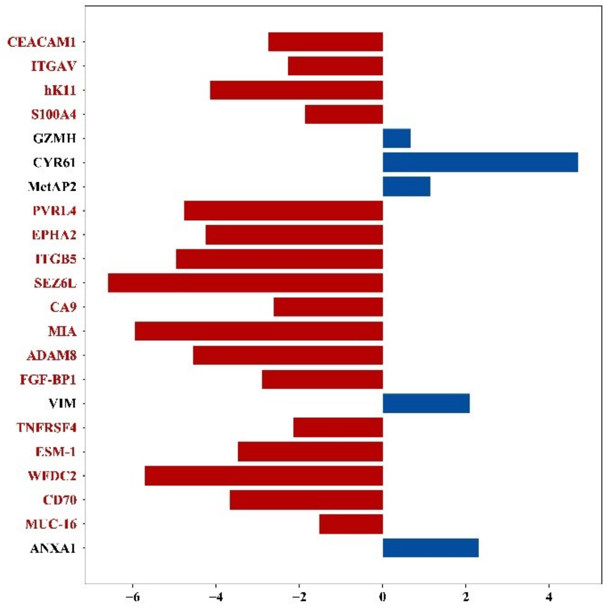

B

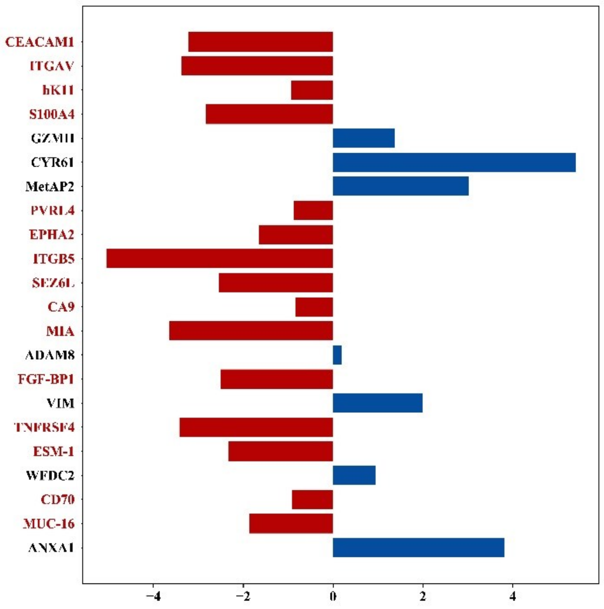

Supplemental Figure 2 Association of cancers with the alternated ANXA1 (A), CYR61 (B), CEACAM1 (C), ITGB5 (D), EPHA2 (E) and VIM (F) protein expression according to GEPIA.

A. ANAX1

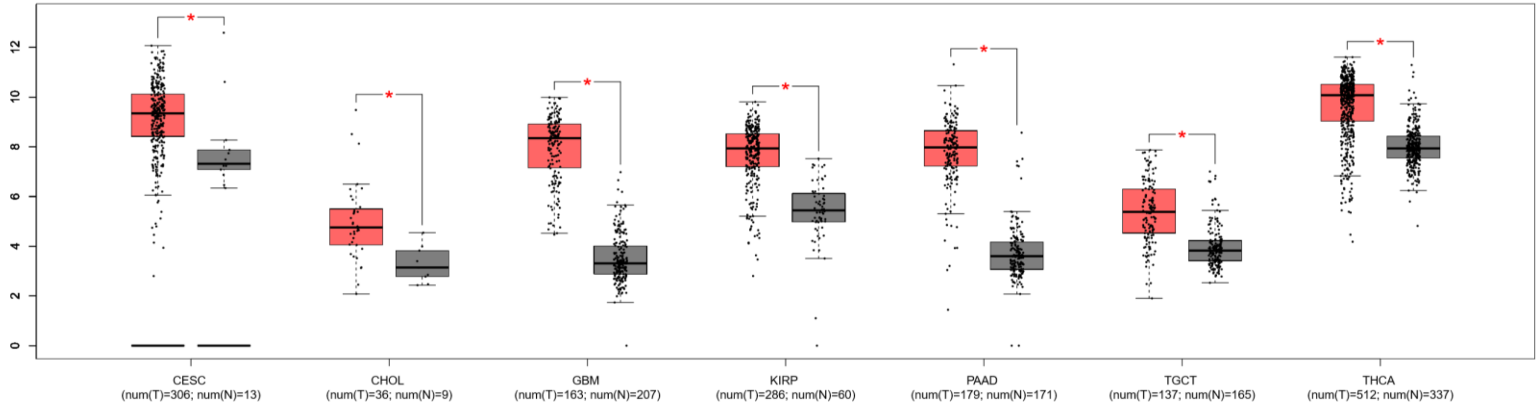

B. CYR61

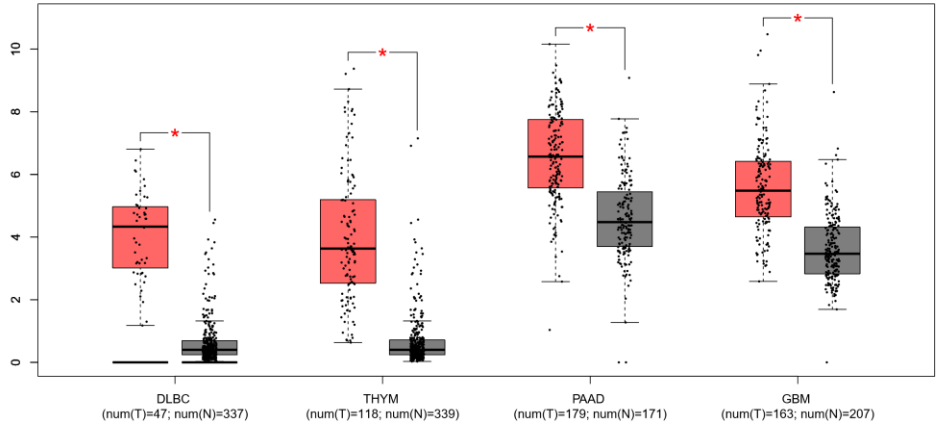

C. VIM

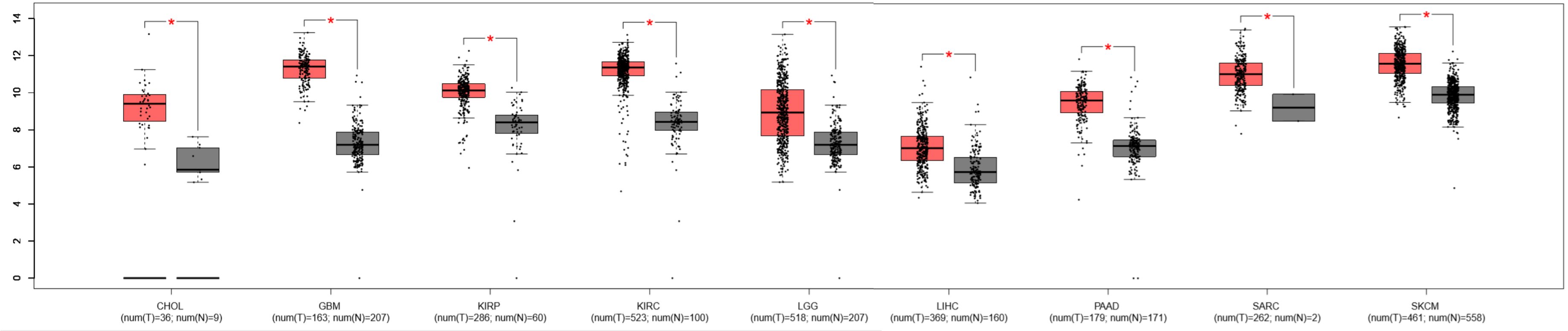

14
12
10
8
6
4
2
0
CHOL
(num(T)=36; num(N)=9)
GBM
(num(T)=163; num(N)=207)
KIRP
(num(T)=286; num(N)=60)
KIRC
(num(T)=523; num(N)=100)
LGG
(num(T)=518; num(N)=207)
LIHC
(num(T)=369; num(N)=160)
PAAD
(num(T)=179; num(N)=171)
SARC
(num(T)=262; num(N)=2)
SKCM
(num(T)=461; num(N)=558)

D. CEACAM1

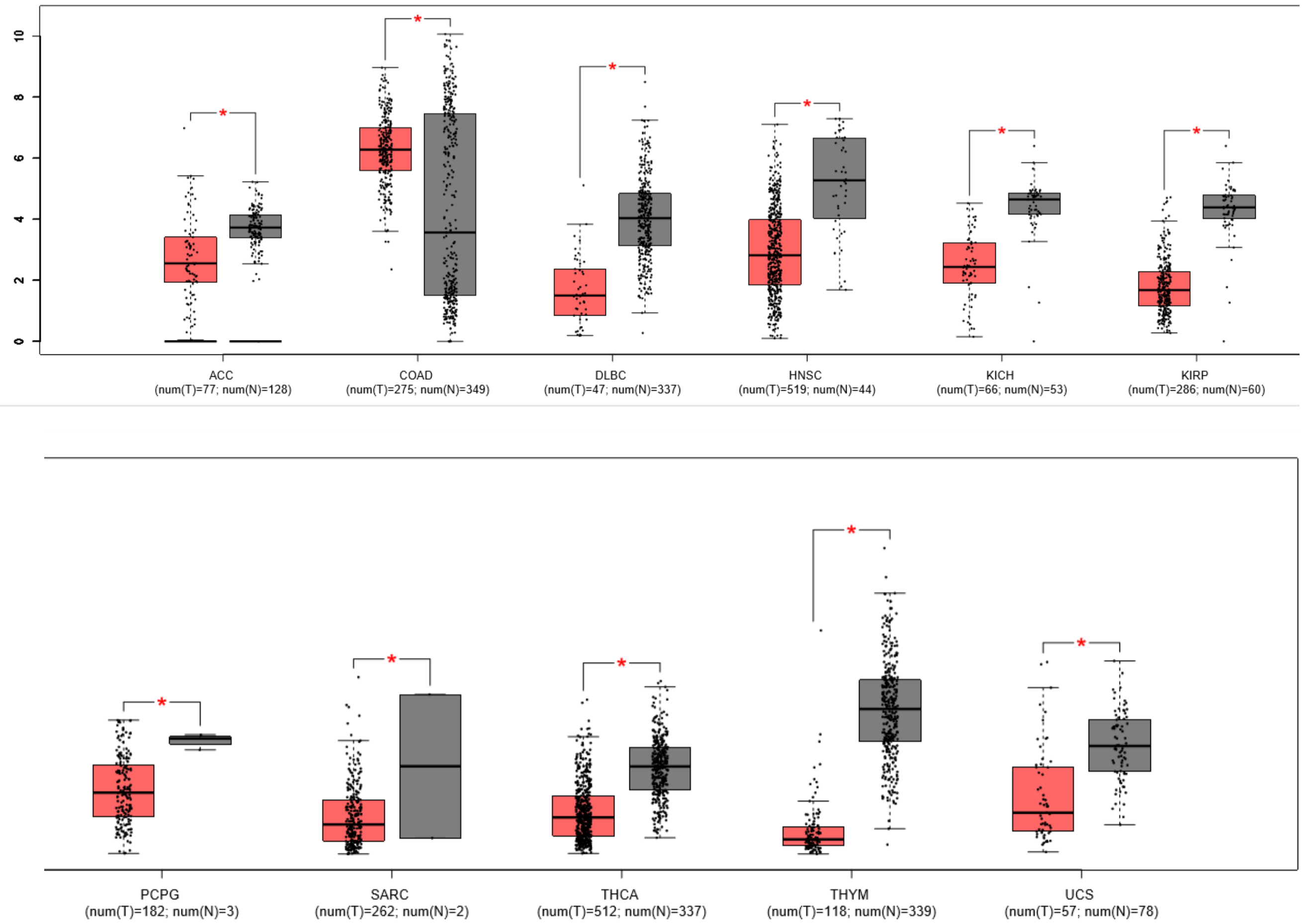
10
8
6
4
2
0
*
ACC
(num(T)=77; num(N)=128)
COAD
(num(T)=275; num(N)=349)
DLBC
(num(T)=47; num(N)=337)
HNSC
(num(T)=519; num(N)=44)
KICH
(num(T)=66; num(N)=53)
KIRP
(num(T)=286; num(N)=60)
PCPG
(num(T)=182; num(N)=3)
SARC
(num(T)=262; num(N)=2)
THCA
(num(T)=512; num(N)=337)
THYM
(num(T)=118; num(N)=339)
UCS
(num(T)=57; num(N)=78)

E. EPHA2

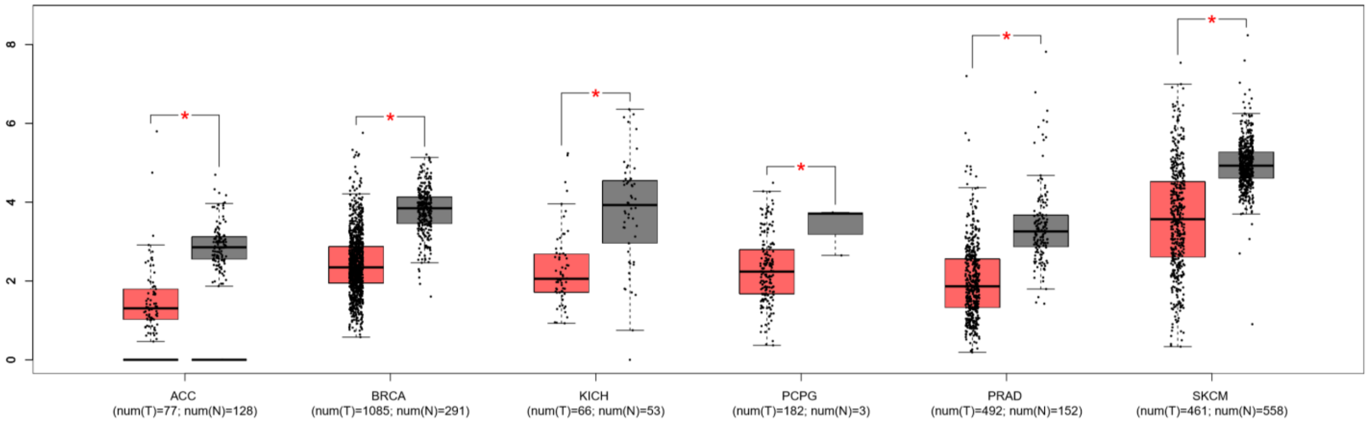

F. ITGB5

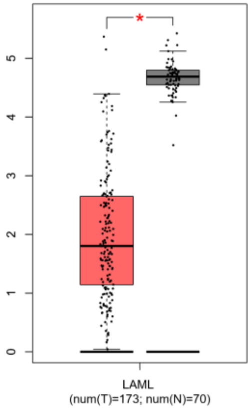

Supplement: Evaluation of Colocasia esculenta Schott in anti-cancerous properties with proximity extension assays [file FNR-65-7549-s001.pdf]
